# The Role of Soluble Urokinase Plasminogen Activator Receptor (suPAR) in the Context of Aneurysmal Subarachnoid Hemorrhage (aSAH)—A Prospective Observational Study

**DOI:** 10.3389/fneur.2022.841024

**Published:** 2022-03-10

**Authors:** Tobias P. Schmidt, Walid Albanna, Miriam Weiss, Michael Veldeman, Catharina Conzen, Omid Nikoubashman, Christian Blume, Daniel S. Kluger, Hans Clusmann, Sven H. Loosen, Gerrit A. Schubert

**Affiliations:** ^1^Department of Neurosurgery, Rheinisch-Westfälische Technische Hochschule (RWTH) Aachen University Hospital, Aachen, Germany; ^2^Clinic for Diagnostic and Interventional Neuroradiology, RWTH Aachen University Hospital, Aachen, Germany; ^3^Institute for Biomagnetism and Biosignal Analysis, University of Münster, Münster, Germany; ^4^Clinic for Gastroenterology, Hepatology and Infectious Diseases, University Hospital Düsseldorf, Medical Faculty of Heinrich Heine University Düsseldorf, Düsseldorf, Germany; ^5^Department of Neurosurgery, Kantonsspital Aarau, Aarau, Switzerland

**Keywords:** delayed cerebral infarction (DCI), subarachnoid hemorrhage (SAH), outcome predictability, inflammation, soluble urokinase plasminogen activator receptor (suPAR)

## Abstract

**Objective:**

Outcome after aneurysmal subarachnoid hemorrhage (aSAH) is highly variable and largely determined by early brain injury and delayed cerebral ischemia (DCI). Soluble urokinase plasminogen activator receptor (suPAR) represents a promising inflammatory marker which has previously been associated with outcome in traumatic brain injury and stroke patients. However, its relevance in the context of inflammatory changes after aSAH is unclear. Here, we aimed to characterize the role of circulating suPAR in both serum and cerebrospinal fluid (CSF) as a novel biomarker for aSAH patients.

**Methods:**

A total of 36 aSAH patients, 10 control patients with unruptured abdominal aneurysm and 32 healthy volunteers were included for analysis. suPAR was analyzed on the day of admission in all patients. In aSAH patients, suPAR was also determined on the day of DCI and the respective time frame in asymptomatic patients. One- and two-sample *t*-tests were used for simple difference comparisons within and between groups. Regression analysis was used to assess the influence of suPAR levels on outcome in terms of modified Rankin score.

**Results:**

Significantly elevated suPAR serum levels (suPAR-SL) on admission were found for aSAH patients compared to healthy controls, but not compared to vascular control patients. Disease severity as documented according to Hunt and Hess grade and modified Fisher grade was associated with higher suPAR CSF levels (suPAR-CSFL). In aSAH patients, suPAR-SL increased daily by 4%, while suPAR-CSFL showed a significantly faster daily increase by an average of 22.5% per day. Each increase of the suPAR-SL by 1 ng/ml more than tripled the odds of developing DCI (OR = 3.06). While admission suPAR-CSFL was not predictive of DCI, we observed a significant correlation with modified Rankin's degree of disability at discharge.

**Conclusion:**

Elevated suPAR serum level on admission as a biomarker for early inflammation after aSAH is associated with an increased risk of DCI. Elevated suPAR-CSFL levels correlate with a higher degree of disability at discharge. These distinct relations and the observation of a continuous increase over time affirm the role of inflammation in aSAH and require further study.

## Introduction

Aneurysmal subarachnoid hemorrhage (aSAH) is characterized by a complex pathophysiology (1–3) and persistently high morbidity and mortality (4). Early brain injury as a direct result of bleeding and delayed cerebral ischemia (DCI) are main contributors to overall outcome, including additional neurological and possibly permanent neurological deficits (5–7). While early brain injury for the most part evades a therapeutical approach, ensuing cascades and complications such as DCI are the focus of most interventional studies. The monocausal concept of angiographic vasospasm as the sole cause of DCI has largely been abandoned, and both clinical and experimental research points to a much more complex nature of the underlying pathophysiology (8, 9). After aSAH onset, a deleterious cascade of cerebral damage and inflammation is initiated that results in a systemic inflammatory state contributing to DCI development (10–12). These inflammatory pathways can promote the formation of microcirculatory spasms, microthrombi, cortical spreading depolarization and disruption of cerebral autoregulation, resulting in a breakdown of normal cellular function and increasing the inflammation itself (8, 13–15). Therefore, the predictive power of several inflammatory markers concerning DCI development has been analyzed, such as IL-6, PCT and leukocyte count (12, 16–20), but has yet failed to translate into clinical relevance.

Soluble urokinase plasminogen activator receptor (suPAR) is a novel and promising, robust inflammatory biomarker, representing the cleavage product of the membrane plasminogen activator receptor (uPAR). While uPAR is expressed on the surface of different cell types, including endothelial and immune cells, suPAR circulates in blood, plasma, serum, cerebrospinal fluid (CSF), and urine (21–23). SuPAR levels are increased in the context of inflammation and thus correlate with the degree of activation of the immune system. Since inflammation is a universal response of the body to various conditions such as tumors, trauma, and ischemia, recent scientific studies investigated the use of inflammatory biomarkers to predict poor outcome. SuPAR has already been shown to be a useful predictor of outcome in patients with neoplasia of the hepatobiliary system and pancreas, as well as neuroendocrine malignancies (24–26). The prognostic significance of suPAR for poor outcome in traumatic brain injury (TBI) and stroke has also been demonstrated (27, 28). Despite the established role of inflammation in the context of SAH, preliminary analyses failed to detect a correlation between suPAR plasma levels and outcome in SAH patients (29). To this end, it is the purpose of this study to further characterize the role of circulating suPAR in serum and CSF as a surrogate for inflammation in aSAH patients.

## Methods

### Ethical Approval and Patient Selection

This is a prospective population-based observational study following registration in https://clinicaltrials.gov (NCT02142166) and approval by the local ethics committee (EK 062/14). The study was performed according to the ethical standards laid down in the 1964 Declaration of Helsinki and its later amendments. Written informed consent was obtained from all patients or their authorized representative. All patients admitted to RWTH Aachen University Hospital with confirmed aSAH between 18 and 90 years of age during the period of 2014 and 2019 were screened for eligibility (*n* = 223). Exclusion criteria were the following: no written consent for study inclusion by the patient or their authorized representative, pregnancy, early mortality within 3 days, non-aneurysmal SAH, mycotic or traumatic aneurysms, as well as aneurysms associated with arteriovenous malformations. For this analysis, only aSAH patients with serum and CSF samples available within 48 h after admission and the day of DCI onset (±24 h) or day 8 (±24 h) as median in patients without DCI were included (*n* = 36). While in 8 patients (6 with DCI, 2 without DCI) the admission samples could be obtained preoperatively, in the remaining 28 samples had to be collected postoperatively. Ten patients with unruptured abdominal aortic aneurysm undergoing elective surgery served as a vascular control group. In the control group, sampling was always performed preoperatively. The healthy control group consisted of 32 volunteers.

### Neurosurgical Treatment Algorithm for SAH Patients at RWTH Aachen University Hospital

All aSAH patients were treated according to a standardized treatment protocol as previously published (30, 31). In summary, ruptured aneurysms were secured by either surgical clipping or endovascular coiling within 48 h after diagnosis of aSAH. Thereafter, all patients were clinically monitored at our dedicated intensive care unit (ICU). Acute hydrocephalus was treated by external ventricular drainage (EVD). All ICU patients received prophylactic nimodipine orally as tolerated (32). In unconscious patients, invasive neuromonitoring was considered as recommended by the 2014 consensus statement (33, 34). Intraparenchymal probes were placed in the border zone of anterior and middle cerebral artery territories: A combined intracranial pressure (ICP) and tissue oxygen partial pressure (p_ti_O_2_) probe (Raumedic AG, Helmbrechts, Germany) as well as a microdialysis catheter (μdialysis, Stockholm, Sweden) for cerebral metabolism parameters such as lactate and glucose.

In awake patients, events of DCI were defined by clinical deterioration using Vangouwen's criteria: new focal neurological deficit or a decrease in GCS ≥ 2 for at least one hour that is not attributable to other diagnoses (5). In unconscious patients, CT perfusion imaging was usually triggered by a new metabolic or oxygenation crisis as determined by either microdialysis (lactate/pyruvate ratio ≥ 40) or p_ti_O_2_ measurements (p_ti_O_2_ <10 mmHg). Classical territorial or watershed hypoperfusion confirmed the diagnosis of DCI. First-line treatment for DCI was euvolemic arterial hypertension ≥ 180 mmHg induced by intravenous noradrenaline infusion. In refractory cases, patients were considered for endovascular rescue treatment by either transluminal balloon-angioplasty or intra-arterial spasmolysis with nimodipine, depending on the localization and distribution of spastic vessels (30).

#### Study Groups

Thirty-six aSAH patients (female: *n* = 32, 88.9%), 32 healthy volunteers (female: *n* = 9, 28.1%), and 10 vascular control patients undergoing elective abdominal aneurysm surgery (female: *n* = 3, 30.0%) were included in this study. Demographic data were obtained including age, sex and BMI. Initial disease severity was assessed at admission according to Hunt and Hess (HH) score and modified Fisher (mF) scale. Aneurysm characteristics and neurological outcome at 6 months are summarized in Table 1 together with the above mentioned baseline data. As the control group of healthy volunteers was recruited in the context of outpatient blood donation, the age is significantly lower compared to the vascular control patients and SAH patients.

**Table 1:** Baseline characteristics of the study groups.

**Characteristic**	**Healthy volunteers**	**Vascular control patients (TAAA)**	**SAH patients**
Age in years-mean ± SD	44 ± 15	56 ± 9	57 ± 14
Sex-no. (%)			
Male	23 (72%)	7 (70%)	4 (11%)
Female	9 (28%)	3 (30%)	32 (89%)
BMI-mean ± SD	26 ± 9	26 ± 9	26 ± 12
Aneurysm location-no. (%)			
MCA	–	–	8 (22%)
Acomm	–	–	15 (42%)
Pcomm and AchA	–	–	3 (8%)
BA	–	–	4 (11%)
Others	–	–	6 (17%)
Aneurysm diameter in mm-mean ± SD	–	–	8.0 ×7.0 ± 5.9 ×3.9
Treatment modality-no. (%)			
Microsurgical	–	–	11 (31%)
Interventional	–	–	25 (69%)
Hunt and Hess grade-no. (%)			
Grade 1	–	–	4 (11%)
Grade 2	–	–	14 (39%)
Grade 3	–	–	11 (31%)
Grade 4	–	–	7 (19%)
Grade 5	–	–	0
Modified fisher grade-no. (%)			
Grade 1	–	–	7 (19%)
Grade 2	–	–	4 (11%)
Grade 3	–	–	9 (25%)
Grade 4	–	–	16 (44%)
mRS 6 months-no. (%)			
Grade 0	32	10	8 (22%)
Grade 1	0 (0%)	0 (0%)	4 (11%)
Grade 2	0 (0%)	0 (0%)	2 (6%)
Grade 3	0 (0%)	0 (0%)	8 (22%)
Grade 4	0 (0%)	0 (0%)	1 (3%)
Grade 5	0 (0%)	0 (0%)	1 (3%)
Grade 6	0 (0%)	0 (0%)	9 (25%)
No data	0 (0%)	0 (0%)	3 (8%)

Average body-mass-indices (BMI) of all three groups were comparable (*p* > 0.64 for all pairwise *t*-tests), as was the average age of aSAH and vascular control patients [*t*_(24. 73)_ = −0.70, *p* = 0.49]. The group of healthy volunteers was significantly younger than both the aSAH [*t*_(28. 81)_ = −3.77, *p* < 0.001] and the vascular control patients [*t*_(52.64)_ = −4.61, *p* < 0.001].

#### Sampling and Data Collection

Since no CSF samples were available from healthy volunteers due to a risk-benefit analysis of a lumbar puncture, a comparison of CSF values was limited to aSAH and vascular control patients. A respective sample collection always provided for simultaneous collection of serum and CSF per day.

Serum and CSF samples were collected and consistently stored at −80°C until the time of analysis. Levels of suPAR were measured using a commercial enzyme-linked immunosorbent assay (ELISA) according to the manufacturer's instructions (NR. A001, suPARnostic, ViroGates, Birkerød, Denmark). The following analyses were based on samples taken on the day of admission (+48 h) and on the day of DCI (±24 h). In patients without DCI, samples of the respective time frame (day 8 ± 24 h after ictus) were analyzed. DCI events and clinical outcome (modified Rankin scale, mRS) were assessed directly after discharge by an independent investigator, and via patient visit or telephone interview after 6 months (35). Neurological outcome was dichotomized by modified Rankin scale (mRS) 0–2 and mRS 3–6.

### Statistical Analysis

Patients and healthy volunteers with any missing data points were excluded prior to statistical analyses. One- and two-sample *t*-tests were used for simple difference comparisons within and between groups, respectively. A regression analysis was constructed to assess the influence of suPAR levels and additional clinical predictors on distinct outcome criteria within the aSAH group taking into account suPAR-SL and suPAR-CSFL, disease severity upon admission (HH, mF), and demographic information (age, sex, BMI) as predictors for the development of DCI. To assess the goodness-of-fit, we tested the logistic regression model (including serum/CSF suPAR levels, questionnaire scores, and demographic variables) against an intercept-only alternative. The corresponding χ^2^-test confirmed or rejected the higher fit of the full model. Subgroup analyses were performed with dichotomized HH score (HH1+2 and HH3–5 for good and poor grade, respectively) and mF scale (mF1+3 and mF2+4 for aSAH with and without intraventricular hemorrhage). All statistical analyses were performed in R Studio (36) and based on a significance level of α = 0.05.

## Results

### suPAR on Admission: Comparison of aSAH Patients With Healthy Controls and Vascular Control Patients

**Figure 1:**
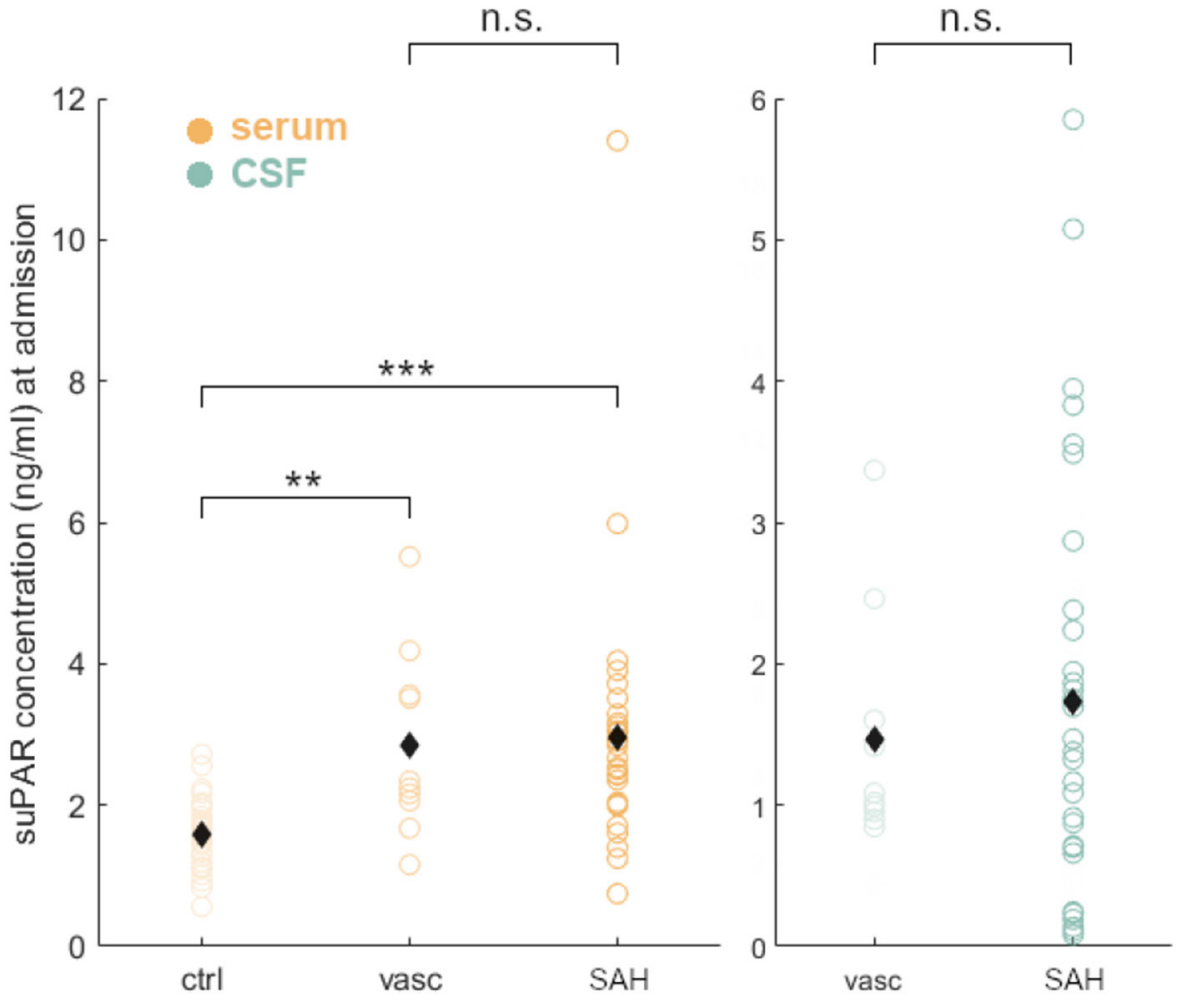
Group comparisons of suPAR serum levels (suPAR-SL, yellow) in healthy volunteers (ctrl), vascular control patients (vasc), and aSAH patients (SAH). Both groups, vasc and SAH, showed a significant higher suPAR concentration compared to ctrl, but did not differ significantly between them in serum and CSF (green). Black diamonds indicate mean values. **: *p* < .01; ***: *p* < .001.

suPAR serum levels (suPAR-SL) on admission were significantly higher in aSAH patients (M = 2.97, SD = 1.84) compared to healthy controls [M = 1.68, SD = 0.44; *t*_(33.69)_ = 3.75, *p* < 0.001; Figure 1], but did not significantly differ between aSAH patients and vascular control patients (*p* = 0.41; Figure 1). Similarly, suPAR CSF levels (suPAR-CSFL) on admission were comparable between aSAH patients and vascular controls (*p* = 0.24; Figure 1). Within the group of aSAH patients, suPAR levels at admission were significantly higher in the serum than in the CSF compartment [*t*_(30)_ = 3.11, *p* < 0.01].

### suPAR on Admission: Comparison of Values in aSAH Patients With Baseline Demographics

Admission suPAR levels did not correlate with age (suPAR-SL: *p* = 0.77; suPAR-CSFL: *p* = 0.10) nor with BMI (suPAR-SL: *p* = 0.99; suPAR-CSFL: *p* = 0.39). Similarly, suPAR-CSFL in male patients at admission were not significantly different from those in female patients (*p* = 0.14).

### suPAR on Admission: Comparison of Values in aSAH Patients With Disease Severity

**Figure 2:**
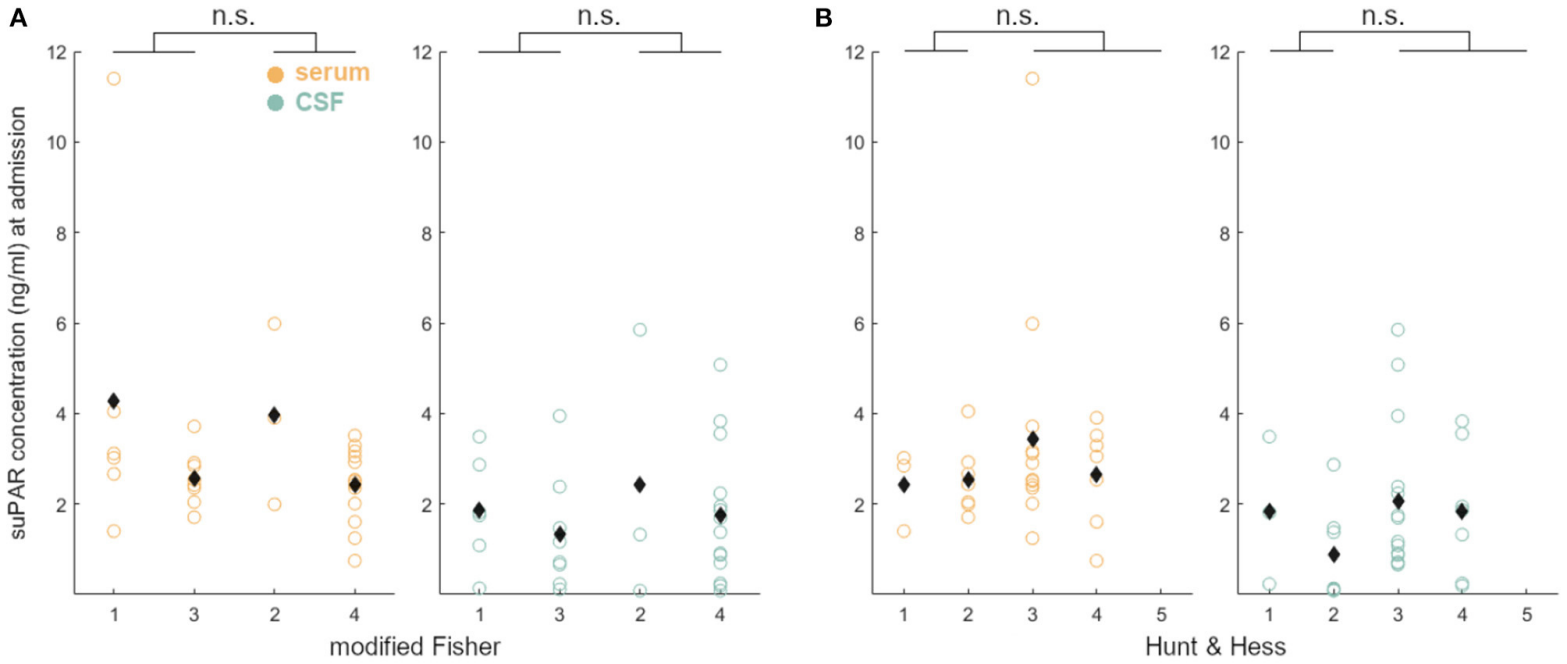
suPAR elevations in aSAH patients on admission did not correlate with disease severity (**A**: mF; **B**: HH).

Neither suPAR-SL (*p* = 0.80) nor suPAR-CSFL (*p* = 0.28) on admission were significantly correlated with the mF scoring within the group of aSAH patients (Figure 2A). Similarly, there was no significant correlation between suPAR-SL (*p* = 0.11) or suPAR-CSFL (*p* = 0.06) at admission and the HH scoring within aSAH patients (Figure 2B).

### Time Course of suPAR Concentrations of aSAH Patients

**Figure 3:**
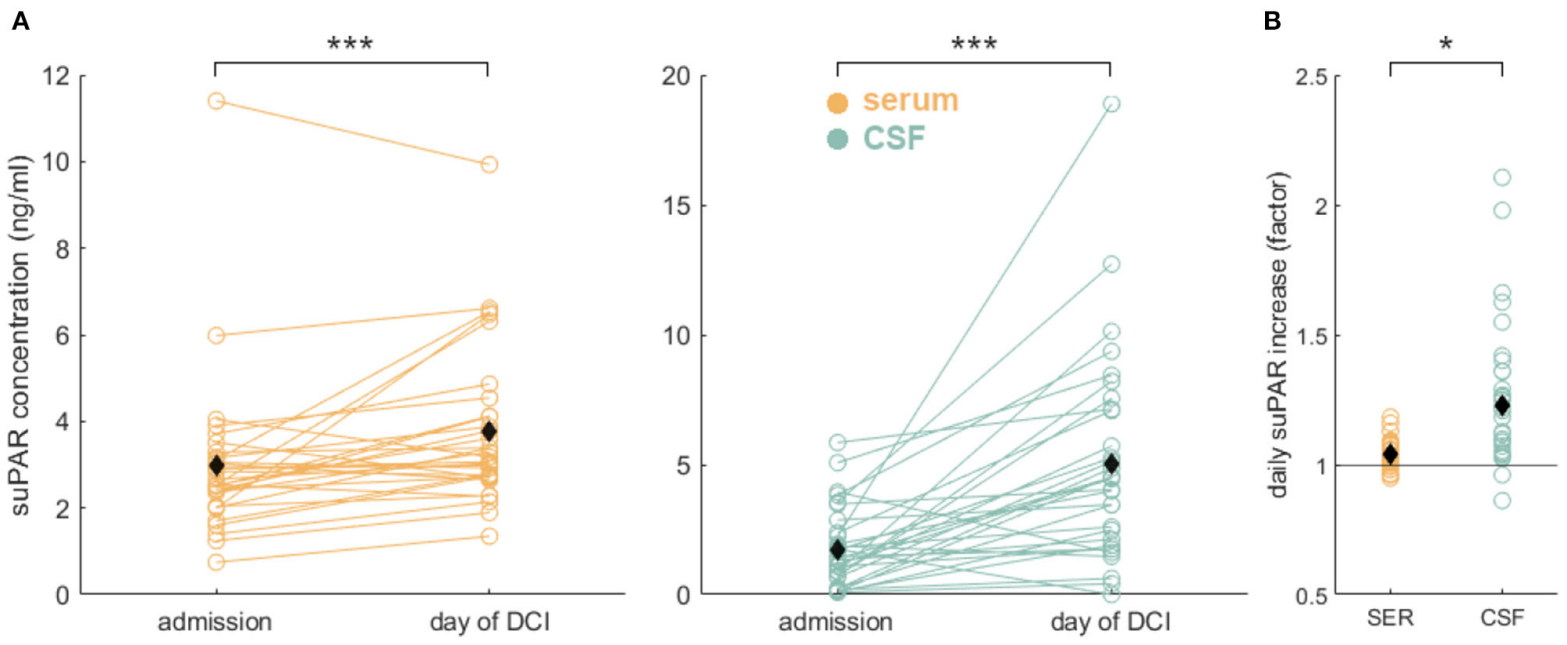
Significant suPAR dynamics in aSAH patients: **(A)** During the hospital stay from admission to the day of DCI or to day 8 for patients without DCI, we detected a significant increase of suPAR-SL (yellow) and for suPAR-CSFL (green). **(B)** Comparison of relative daily suPAR increase in serum (SER) compared to CSF. We found a significantly higher daily increase in CSF compared to serum. Black diamonds indicate mean values. *: *p* < .05; ***: *p* < .001.

Both suPAR-SL (M_diff_ = 0.78, SD = 1.28, *p* = 0.002) and suPAR-CSFL (M_diff_ = 3.29, SD = 3.80, *p* < 0.001) increased significantly over time (Figure 3A). suPAR-SL on average increased by 4% (SD = 0.56%) each day, while suPAR-CSFL increased significantly faster by an average of 22.5% daily [SD = 3.6%, *t*_(30)_ = 2.66, *p* = 0.01; Figure 3B].

### suPAR on Admission: Association of Values in aSAH Patients With DCI, Cerebral Infarction, and Neurological Outcome

**Figure 4:**
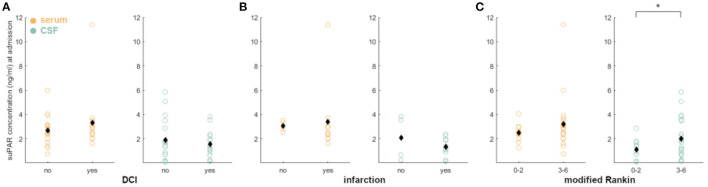
Correlation of admission suPAR levels with DCI, DCI-related infarction and neurological outcome: **(A)** There were no significant differences, when correlating suPAR-SL and suPAR-CSFL with DCI occurrence. **(B)** The same is true for the correlation with DCI-related infarctions. **(C)** Dichotomizing the patients in good outcome (mR 0–2) and poor outcome (mR 3–6), we detected a significant correlation with outcome early elevated suPAR-CSFL (green) but not suPAR-SL. No significant difference was found for outcome and mortality after 6 months (data not shown). Black diamonds indicate mean values. *: *p* < .05.

Higher levels of suPAR-SL (β = 1.12, *z* = 2.08, *p* = 0.04) on admission showed significant predictive effects for the occurrence of DCI. More specifically, each increase of the suPAR-SL by 1 ng/ml more than tripled the odds of developing DCI (OR = 3.06). We confirmed the significantly higher fit of the full model [$\chi_{\left(8\right)}^{2}$ = 16.52, *p* = 0.04]. aSAH patients without DCI had no significant difference between suPAR-SL and suPAR-CSFL on admission (*p* = 0.14). In contrast, aSAH patients with DCI showed a significantly higher suPAR-SL compared to suPAR-CSFL [suPAR-SL: M = 3.30, SD = 2.41; suPAR-CSFL: M = 1.54, SD = 1.17, *t*_(18.81)_ = 2.46, *p* = 0.02]. Neither suPAR-SL (*p* = 0.40) nor suPAR-CSFL (*p* = 0.14) on admission correlated with the development of DCI related infarctions (Figure 4A).

aSAH patients without DCI-related cerebral infarction had no significant difference between suPAR-SL and suPAR-CSFL on admission (*p* = 0.05; Figure 4B). The same was evident for aSAH patients with DCI-related cerebral infarction (*p* = 0.41; Figure 4B).

Early suPAR-SL levels did not correlate with outcome (*p* = 0.10; Figure 4C), while elevated suPAR-CSFL was associated with worse neurological outcome [mR 0–2 at discharge: M = 1.11, SD = 0.91; mR 3–6: M = 2.02, SD = 1.67, *t*_(28.34)_ = −1.96, *p* = 0.03; Figure 4C]. Neither suPAR-SL (*p* = 0.36) nor suPAR-CSFL (*p* = 0.81) on admission correlated with neurological outcome after 6 months nor with mortality after 6 months (suPAR-SL *p* = 0.98; suPAR-CSFL *p* = 0.32).

### suPAR at the Time of DCI: Comparison of Values in aSAH Patients With Disease Severity

**Figure 5:**
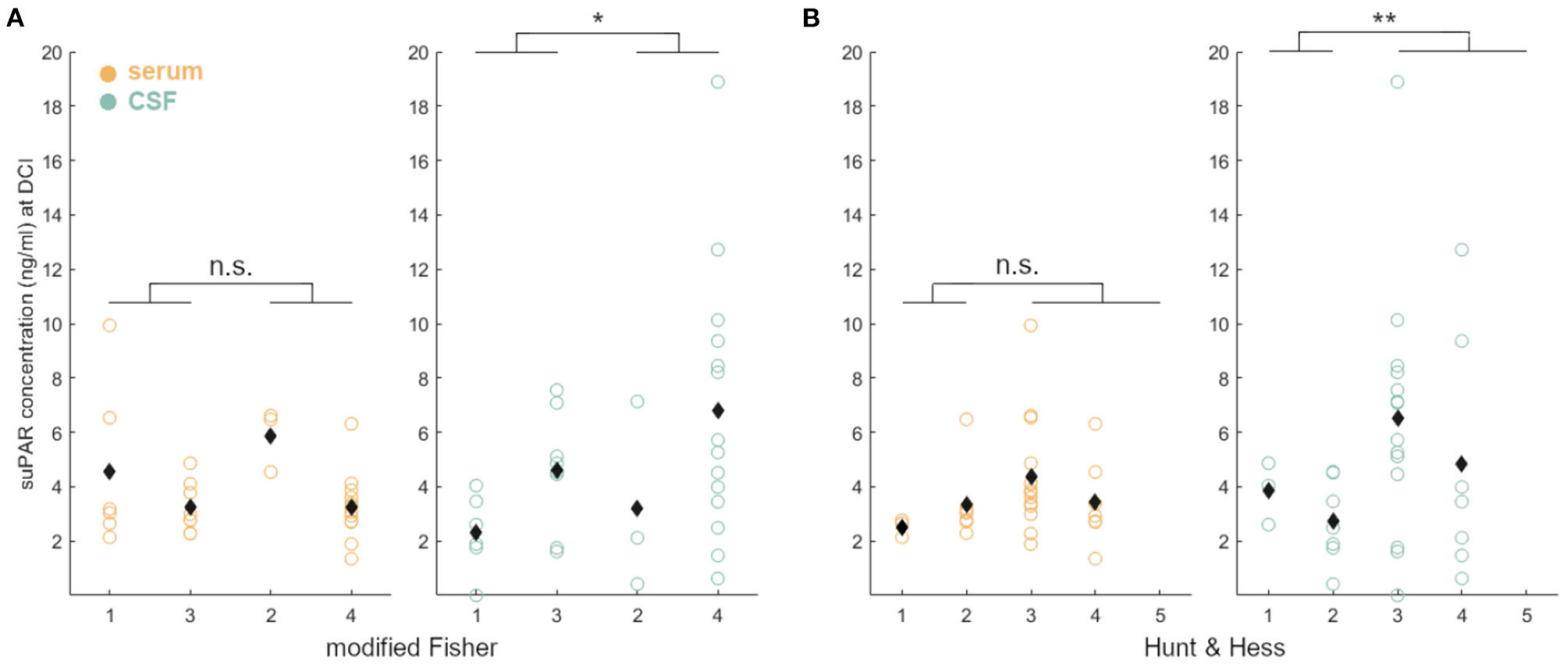
Correlation of disease severity and suPAR elevations on the day of DCI: suPAR-SL (yellow) levels did not correlate with radiographic or clinical severity (**A**: mF, **B**: HH). However, elevated suPAR-CSFL (green) correlated significantly with the presence of intraventricular hemorrhage and more severe neurological deficit. Black diamonds indicate mean values. *: *p* < .05; **: *p* < .01.

There was no correlation between suPAR-SL and severity of hemorrhage (*p* = 0.56; Figure 5A), but suPAR-CSFL was significantly higher in aSAH patients with intraventricular blood [mF 1+3: M = 3.62, SD = 2.17; mF 2+4: M = 6.17, SD = 4.83, *t*_(23.10)_ = −1.95, *p* = 0.03; Figure 5A]. Serum suPAR was comparable in patients with good grade and poor grade SAH (*p* = 0.06), while CSF suPAR was significantly higher in aSAH patients with high grade HH [HH 1–2: M = 3.06, SD = 1.46; HH 3–5: M = 5.96, SD = 4.51, *t*_(26.90)_ = −2.66, *p* = 0.006; Figure 5B].

### suPAR at the Time of DCI: Association of Values in aSAH Patients With DCI, Cerebral Infarction, and Neurological Outcome

**Figure 6:**
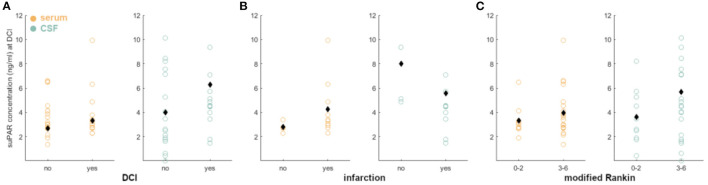
Correlation of suPAR elevations in aSAH patients at the day of DCI with DCI, DCI-related infarction and neurological outcome: **(A–C)** We detected no significant difference in suPAR-SL and suPAR-CSFL in patients with and without DCI, DCI-related infarction, or neurological outcome. Black diamonds indicate mean values.

aSAH patients without DCI had no significant difference between late suPAR-SL and suPAR-CSFL (*p* = 0.73; Figure 6A). The same was evident for aSAH patients with DCI (*p* = 0.09; Figure 6A). aSAH patients without DCI-related cerebral infarction had no significant difference between late suPAR-SL and suPAR-CSFL (*p* = 0.95; Figure 6B). The same was true for aSAH patients with DCI-related cerebral infarction (*p* = 0.05; Figure 6B). SuPAR levels at the time of DCI were not able to predict outcome at the time of discharge (suPAR-SL: *p* = 0.14; Figure 6A; suPAR-CSFL: *p* = 0.05; Figure 6C) and not at 6 months (suPAR-SL: *p* = 0.10; suPAR-CSFL: *p* = 0.203). No statistical significance was found in terms of mortality at 6 months for both suPAR-SL (*p* = 0.99) and suPAR-CSFL (*p* = 0.083).

## Discussion

The development of DCI is strongly associated with the risk of poor outcome after aSAH, but the prediction of DCI and poor outcome poses a major challenge for the lack of robust biomarkers (37, 38). Neuroinflammatory cascades may lead to DCI and DCI-related infarctions (12, 16, 20), typically occurring with a characteristic latency after aSAH (day 4–14). As inflammation plays a crucial role in DCI pathogenesis, novel, rapidly responding neuroinflammatory biomarkers may enable an early identification of high risk patients (39, 40). suPAR is a very sensitive biomarker in sepsis, TBI and ischemic stroke concerning morbidity and mortality, making it a promising candidate in the context of SAH (27, 28, 41, 42). Thus, we examined the role of suPAR in serum and in CSF in aSAH patients.

We found that suPAR-SL on admission was elevated in aSAH patients compared to healthy controls (Figure 1), indicating a systemic, possibly aSAH-related, early inflammatory response. Interestingly, no significant difference could be detected between the vascular control group and the aSAH group at the time of admission in both serum and CSF which may in part be explained by the presence of chronic inflammation known to occur in unruptured abdominal and intracranial aneurysms (43–46). As a consequence, it remains elusive whether intracranial aneurysm rupture (as a local event with an immediate systemic inflammatory increase) or the preexisting condition of chronic inflammation is causative of higher suPAR levels. However, it is conceivable that a local, compartmentalized inflammatory processes within the cerebral aneurysm wall are probably not detectable systemically, rendering an early inflammatory response caused by the rupture more likely (47). An alternative hypothesis may be that chronic, low grade, but systemic inflammation may support or even initialized the formation of aneurysm (48). A future comparison of suPAR levels in patients with unruptured cerebral aneurysms may help to clarify this relationship. Neuroinflammatory recruitment to the CSF space appears to be important for the relationship between systemic inflammation and organ-systemic vascular complications in the brain of aSAH patients. Thereby, the integrity of the blood-brain barrier plays a major role, although with this study it remains unclear whether suPAR can enter the subarachnoid space secondary to a disruption of the blood-brain barrier or whether it is produced more abundantly in this space (49). Further studies are needed to address this topic.

**Figure 7:**
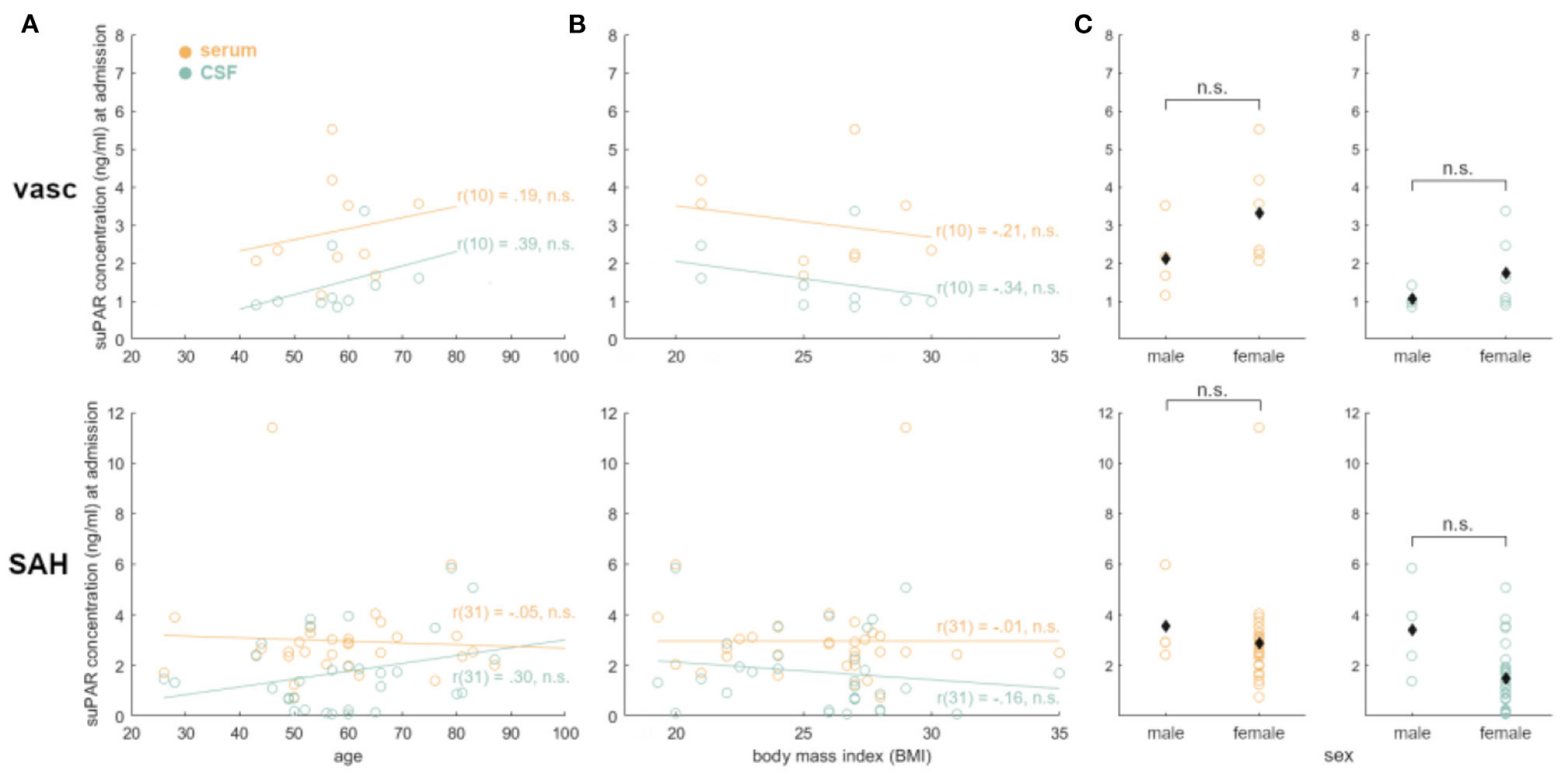
suPAR elevations in aSAH patients were not affected by baseline characteristics: no statistical significance was found for age **(A)**, BMI **(B)**, oder sex **(C)**. suPAR-SL in yellow. suPAR-CSFL in green. Black diamonds indicate mean values.

According to our data, neither suPAR-SL nor suPAR-CSFL on admission correlated with the patient's baseline characteristics such as age, BMI and sex (Figure 7). Furthermore, both suPAR levels on admission showed no correlation with the initial modified Fisher (mF) as well as Hunt and Hess (HH) severity scores (Figure 2). Over time, suPAR-SL increased by an average of 4% per day, while suPAR-CSFL increased significantly faster by an average of 22.5% daily thus implying a more accentuated local response (Figure 3). This could be due to a recruitment of neuroinflammatory cells and molecules into the CSF compartment after aSAH onset, triggering inflammatory cascades (50, 51). At the time of DCI, disease severity (mF 2+4; HH 3–5) was mirrored in higher levels of suPAR (Figure 5).

Higher levels of suPAR-SL on admission were predictive for the occurrence of DCI: each increase of the suPAR-SL by 1 ng/ml more than tripled the odds of developing DCI (OR = 3.06) but failed to predict outcome at 6 months. Although we detected a significant predictive effect for admission suPAR-SL regarding the occurrence of DCI, it should be noted that this effect is at least in part driven by one aSAH patient with a particularly high suPAR concentration. This patient suffered from a ruptured aneurysm of the communicating anterior artery with initial hemiplegia and epileptic seizure (HH 3 and mF 2). suPAR levels both on the day of admission and on the day of DCI were significantly elevated, rendering a single aberrant reading unlikely. Because of this outlier affecting our statistical analysis, our analysis unfortunately fails to confirm or profoundly refute the results of Kiiski et al. regarding the predictive power of suPAR in serum, calling for future studies to replicate this effect in larger samples (29).

To our knowledge, this is the first study analyzing the role of suPAR in the CSF. The neuroinflammatory response after aSAH is highly complex with many cross-linked processes within and between the blood and CSF compartments. The analysis of a single biomarker in a single compartment does not do justice to this complexity, nor is it likely to yield a single biomarker for prediction. Whether serum biomarkers are sufficiently sensitive, in particular compared to biomarkers in the CSF compartment, needs to be determined. However, the vast heterogeneity of clinical courses requires a more tailored and individualized treatment approach, which potentially could be guided by systemic or local surrogate markers. And indeed, we detected a significant correlation of elevated suPAR-CSFL on admission with poor neurological outcome at discharge. However, this correlation was no longer significant for either morbidity nor mortality at 6 months, and at this time limits the practical relevance of suPAR to the immediate clinical course.

Our study is limited by its monocentric design and a selection bias due to the restriction of subgroup analyses to aSAH patients with existing serum and CSF samples. The statistical power was reduced, particularly in subgroup analysis. Consequently, because our observations are based on a subgroup of aSAH patients in our prospective cohort, they should be considered only as hypothesis-generating. Another limitation is the timing of serum and CSF sampling. While all samples of the control groups were obtained before any sort of invasive procedure, a significant proportion of early samples in the SAH group were obtained only after the intervention (EVD placement, clipping, coiling) which may have skewed the data in terms of group comparison, but only to a lesser extent the subgroup analysis of aSAH patients. The DCI definition remains a major challenge and, in our opinion, is still currently neither standardized nor unified in language. Although Vergouwen's work from 2010 pointed out the inconsistent use of the DCI term in clinical trials and called for a standardized clinical definition and language, this standardization has not yet been completed. Therefore, we specifically note the limitation of our study, which refers to the DCI definition used in our department as described in the Materials and Methods section above.

In summary, our study reports first data on the neuroinflammatory marker suPAR in serum and CSF in the acute phase of aSAH. It may be hypothesized that elevated suPAR-CSFL reflect inflammation in the CNS, associated with a higher risk for vascular complications such as DCI, which in turn may further aggravate neuroinflammation and ultimately result in a poor outcome. Future studies should examine these dynamics of suPAR-CSF concentrations during the acute phase of SAH in more detail to confirm the diagnostic role of suPAR in secondary disease progression.

### Conclusion

Early elevation of serum suPAR is associated with an aggravated clinical course after aSAH, while early elevation in the CSF correlates with a higher risk of poor outcome. Though the exact causal relationship remains to be determined, suPAR may serve as a novel neuroinflammatory biomarker after aSAH.

## Data Availability Statement

The raw data supporting the conclusions of this article will be made available by the authors, without undue reservation.

## Ethics Statement

The studies involving human participants were reviewed and approved by Ethics Committee at the Medical Faculty of RWTH Aachen University (RWTH Aachen), Universitätsklinikum Aachen Pauwelsstraße 30 52074 Aachen. The patients/participants provided their written informed consent to participate in this study.

## Author Contributions

TS, SL, and GS conceived and designed the study. WA, MW, and CC organized the database. DK performed statistical analyses. TS wrote the first draft of the manuscript draft. MV, ON, CB, and HC wrote sections of the manuscript. All authors contributed to manuscript revision, read, and approved the submitted version.

## Conflict of Interest

The authors declare that the research was conducted in the absence of any commercial or financial relationships that could be construed as a potential conflict of interest.

## Publisher's Note

All claims expressed in this article are solely those of the authors and do not necessarily represent those of their affiliated organizations, or those of the publisher, the editors and the reviewers. Any product that may be evaluated in this article, or claim that may be made by its manufacturer, is not guaranteed or endorsed by the publisher.
